# Analyzing activity and injury risk in elite curling athletes: seven workload monitoring metrics from session-RPE

**DOI:** 10.3389/fpubh.2024.1409198

**Published:** 2024-08-13

**Authors:** Junqi Wu, Fan Zhao, Chunlei Li

**Affiliations:** ^1^Beijing Sport University, Beijing, China; ^2^Beijing Research Institute of Sports Science, Beijing, China

**Keywords:** session-RPE, injury classification, neural network, workload, injury risk

## Abstract

**Objective:**

The study aimed to compare the differences in the performance of seven session-rating of perceived exertion (RPE)-derived metrics (coupled and uncoupled acute: chronic workload ratio (ACWR), weekly ratio of workload change, monotony, standard deviation of weekly workload change, exponentially weighted moving average (EWMA), and robust exponential decreasing index (REDI)) in classifying the performance of an injury prediction model after taking into account the time series (no latency, 5-day latency, and 10-day latency).

**Design:**

The study documented the RPE of eight curlers in their daily training routine for 211 days prior to the Olympic Games.

**Methods:**

Seven Session-RPE (sRPE)-derived metrics were used to build models at three time series nodes using logistic regression and multilayer perceptron. Receiver operating characteristic plots were plotted to evaluate the model’s performance.

**Results:**

Among the seven sRPE-derived metrics multilayer perceptron models, the model without time delay (same-day load corresponding to same-day injury) exhibited the highest average classification performance (86.5%, AUC = 0.773). EMWA and REDI demonstrated the best classification performance (84.4%, *p* < 0.001). Notably, EMWA achieved the highest classifying accuracy in the no-delay time series (90.0%, AUC = 0.899), followed by the weekly load change rate under the 5-day delay time series (88.9%, AUC = 0.841).

**Conclusion:**

EWMA without delay is a more sensitive indicator for detecting injury risk.

## 1 Introduction

RPE was first pioneered by Borg in the 1960s and 1970s to engage in research related to the perception of physical exertion, and he also proposed the 6–20 scale, the CR-10 scale, and the CR-100 scale (1). The study by Banister et al. proposed a stimulus-fatigue model on exercise load and the concept of training impulse (TRIMP) and quantified and monitored internal load characteristics in various competitive sports based on HR (2). Researcher Foster, influenced by the studies of the previous two scholars, optimized the CR-10 scale and proposed Foster’s modified version of the CR-10 RPE scale, which is currently the most widely used in competitive sports (3). In the subsequent studies, he proposed the sRPE load monitoring method, which quantifies the training/competition load by monitoring the duration of the training/competition combined with the RPE after training/competition, in which session refers to the duration of training/competition, i.e., Work Load of training/competition = RPE × Session Duration(min), A.U. (Arbitrary Units) (4). sRPE is a method to monitor the average intensity of training/competition, which is of great practical value and provides conditions for the effective quantification of exercise load (5).

ACWR is the most commonly used indicator for assessing injury risk in the practice of sRPE, with ACWR between 0.8 and 1.3, athletes are at low risk of injury, and with ACWR above 1.5, the risk of injury is significantly increased (6), and Gabbett suggests that with ACWR between 0.8 and 1.3, athletes are at low risk of injury and are in a “green zone” (7), and with ACWR above 2, the non-contact injury rate increases by 5–6 times (7). Gabbett suggested that athletes with an ACWR of 0.8–1.3 have a low risk of injury and are in a “green health zone” (7). He also noted that the rate of non-contact injuries increases by a factor of 5–6 when the ACWR exceeds 2 (8) and that ACWR is significantly correlated with the risk of non-contact injuries, but that program variations make it impossible to determine the ACWR threshold for the smallest risk of injury (9).

The value of the coupled ACWR for use during training is worth determining, but its reliability has been questioned. The coupled ACWR is a ratio of activity loads and is not a measure of change, while simply going through the ratio to normalize acute loads into chronic loads is mathematically inaccurate and is required to be predicated based on the existence of a linear relationship between the numerator and denominator in the ratio (10). The essence of this is that acute load is higher than past chronic load and injury risk increases, and excessively lower than chronic load and injury risk increases. However, there are limitations in that the coupled ACWR creates a spurious correlation between acute and chronic loads, resulting in a reduction in interval workload variability. Many athletes will adjust their athletic status and recover from high-intensity training by decreasing the amount of training prior to the competition. The performance of ACWR will differ significantly from the actual situation; there are a large number of confounding factors, similar to the pre-competition reduction in the actual process, which results in a forced decrease in load, causing a rapid change in the tendency of the ACWR, but there is no risk of injury (11). Otherwise, there is sometimes a lag in the occurrence of injury, i.e., an injury from an overloaded training session in the current week may occur in the following week, and this injury is calculated into the load of the following week in the algorithm of the coupled ACWR, resulting in a decrease in sensitivity (12). In addition, the average loading calculation ignores intensity stimuli during the loading effect and reduces the effect of training intensity peaks on the loading effect over time. Conversely, the weighted loading approach, which increases the weight of recent training through the weighting factor, has the disadvantage of being particularly sensitive to missing data halfway through the process (13).

Therefore, different researchers have proposed different coping strategies based on their studies to address the above-mentioned problems. Researcher Lolli supports the effectiveness of uncoupled ACWR in improving variability, i.e., not including acute loads in rolling loads to increase their variability (14). Researcher Williams refined the problem of load time series accumulation by proposing an algorithm for load weighting of sRPE decaying over time, EWMA (11). Researcher Montini, based on Williams’ study, added a lag perspective to correspond the previous week’s damage to the current week’s load, and the findings affirmed the correlation between lagged damage and load (12). Researcher Moussa introduced a natural logarithmic weight, the REDI, to optimize the negative impact of missing data on the overall data analysis (13). Researchers Lazarus and Tysoe, on the other hand, used a new tool, the rate of change of weekly load and the standard deviation of weekly load change, to study the relationship between load and injury risk, and the results concluded that when the load increases by 1 standard deviation, it will cause the risk of injury, and when it increases by 2 standard deviations, it will cause a high risk of injury (15, 16). Meanwhile, for the traditional means of risk correlation analysis, the use of artificial neural networks in machine learning to analyze will achieve better results (17). However, there are still many controversies about the effectiveness of the optimization means of ACWR, and coupled and uncoupled ACWR do not seem to have much difference in estimating injury risk (18). The findings of some other researchers affirm that EWMA is more sensitive than ACWR in injury risk association. There are also studies that show REDI captures injury risk better than ACWR and EWMA (19), but REDI’s findings are based on simulated data, and its real-world application is still questioned.

Curling is a sport dominated by technical and tactical skills. On the international stage, the injury rate and severity in curling are relatively low, and the athletic lifespan of curlers is long, making injury management crucial (20). As curlers age in their sport, their experience grows richer, which is critical for a non-contact sport. Injuries in curling are predominantly chronic, often stemming from fatigue accumulation and prolonged abnormal movement patterns (21). The sport’s workload encompasses both psychological and physiological dimensions, making the sources of this load complex and ambiguous, which complicates injury prevention for coaches and team doctors. The integration of computer science into sports science is a direction for the development of competitive sports. Numerous studies currently use machine learning to identify risk factors for injuries. sRPE is a straightforward method for monitoring workload, and if combined with machine learning to optimize its shortcomings, it could better help control injury risks. In Palmer’s research (22), machine learning and sRPE were used to identify sensitive factors for injury risk, but the study did not explore various derivative algorithms of ACWR. Maintaining long-term physical health is vital for curlers. Given their extended athletic lifespan, curling requires a simpler, more convenient, and cost-effective method for workload monitoring, which sRPE can effectively provide.

This study aimed to explore the sensitivity of coupled ACWR, uncoupled ACWR, weekly load change rate, monotony, standard deviation of weekly load change, EWMA, and REDI to capture injury risk at different lags after logistic regression and neural network modeling, and to search for the best ACWR optimization under one optimal lag period means to correlate injury risk, to help the trainer’s training program development and revision process.

## 2 Methods

### 2.1 Participants

The subjects were eight members of the Chinese National Men’s Curling Training Team preparing for the Winter Olympic Games, with an average age of 26.8 years (22–31 years), an average height of 181.1 ± 10.7 years, an average body weight of 77.2 ± 5.5 years, and an average number of years of training of 8.6 ± 2.4 years. All athletes provided an informed consent form. The collection period was 211 consecutive days (Table 1).

**Table 1:** Basic information table of participants in the experiment.

	Position first and second (*n* = 4)	Position third and fourth (*n* = 4)	All (*n* = 8)
Age (year), mean (range)	25.5 (22–25)	28 (25–31)	26.8 (22–31)
height (cm), mean (SD)	182.8 (5.9)	179.5 (3.7)	181.1 (4.9)
weight (kg), mean (SD)	81.3 (1.7)	73.1 (4.8)	77.2 (5.5)
BMI (kg/m^2^), mean (SD)	24.4 (1.2)	22.6 (0.7)	23.5 (1.3)
Skeletal muscle (kg), mean (SD)	40.7 (1.5)	36.4 (2.6)	38.6 (3.0)
Body fat (%), mean (SD)	12.5 (2.1)	12.7 (1.3)	12.6 (1.6)
Training year (year), mean (SD)	7.8 (1.5)	9.5 (1.9)	8.6 (2.4)

### 2.2 RPE collection

The training duration and RPE of the athletes for field training and physical training were collected 15–30 min after the end of training using the Borg CR-10 scale (5). Athletes who had an injury stoppage were scored as 0 A.U. on that day if there was no other form of practice. Training duration was defined in this study as the period from the start of training to the end of training. The “start of training” was defined in this study as the “start of training” when the athletes entered the field to start the official training program developed by the coaches after the uniform standardized warm-up. The “end of training” is defined as the athlete completing the formal training tasks formulated by the coaches and leaving the main training site, which is the end of training, excluding the process of relaxation and stretching after training. Combined with the actual situation of training, the training week is 5 days, the first 4 days for a number of field training and physical training, and the fifth day for rest.

### 2.3 Index calculation

RPE was used in this study using Foster’s modified version of the CR-10 RPE scale (3), and workload (4) was calculated as follows:

Workload=RPE×training duration(min),A.U.(Arbitrary Units)

In ACWR, acute load was recorded as the sum of the daily workload during the last 1 week, and chronic load was recorded as the weekly average of the sum of workload during the last 4 weeks (7). The ACWR was calculated as follows:

$$Coupled\;\;ACWR=\frac{Workload_{1\;\;week}}{Workload_{Average\;\;4\;\;weeks}}$$

In the uncoupled ACWR, acute load was recorded as the sum of the daily workload during the last 1 week, and chronic load was recorded as the weekly average of the sum of the workload during the last 3 weeks (14). The ACWR was calculated as follows:

$$Uncoupled\;\;ACWR=\frac{Workload_{1\;\;week}}{Workload_{Average\;\;3\;\;weeks}}$$

The weekly ratio of workload change is the ratio of the sum of the workload of the last week to the sum of the workload of the previous 1 week (16). The Diff was calculated as follows:

$$Diff=\frac{Workload_{last\;\;week}}{Workload_{previous\;\;week}}$$

Monotony is the ratio of the most recent 1-week workload to the standard deviation of the weekly workload (23). The monotony was calculated as follows:

$$Monotony=\frac{Workload_{last\;\;week}}{WeeklyWorkload_{SD}}$$

The standard deviation of weekly workload change is the difference between the most recent 1-week workload and the average weekly workload, vs. the variance of the weekly workload (15). The SDΔ was calculated as follows:

$$\mathrm{SD}\Delta =\frac{Workload_{last\;\;week}-Week\;\;Workload_{average}}{W\mathrm{eek}\;\;Workload_{\mathrm{var}iance}}$$

EWMA is based on Williams’ study (11) and the actual training period design in this study, with a decay time of *N* = 5. The EWMA ACWR was calculated as follows:

$$EWMA_{today}=Workload_{today}+\left(\left(1-\lambda \right)\times EWMA_{\mathrm{yesterday}}\right)$$

$$\lambda =\frac{2}{N+1},\;\;\lambda \in \left(0,\;\;1\right)$$

$$ACWR_{EWMA}=\frac{EWMA_{1\;\;week}}{EMWA_{\mathrm{average}\;\;4\;\;week}}$$

REDI based on Moussa’s study (13), in this study, taking into account the problem of actual rest days for training and maintaining consistency with the EWMA time-decrement weighting form, the natural logarithmic time-decrement in this study was performed only during the week, without weighting the full training day, and for the rest days occurring due to objectively uncontrollable factors in the training process, the same time-decrement clearing is performed, and time-decrement weighting is reapplied on the following day operations. The REDI ACWR was calculated as follows:

$$REDI_{\mathrm{today}}=\frac{1}{\sum_{i=1}^{N}\alpha_{i}}\times \overset{N}{\underset{i=0}{\sum }}\alpha_{i}\times Workload_{today}$$

$$\alpha_{i}=e^{-i};\mathrm{if workload is missing},\;\;\alpha_{i}=0$$

$$ACWR_{REDI}=\frac{REDI_{1\mathrm{week}}}{REDI_{\mathrm{aver}age4week}}$$

### 2.4 Definition of injury

The present study was based on the International Olympic Committee Joint Statement (24), which statistically defines injury and illness as one complete injury or illness from the time it occurs to the time of full recovery after receiving medical treatment. Injury definition, classification (site, severity, type, etc.), and mechanism were referred to high-confidence statistical studies of sports injuries or the latest official consensus statement (24), and the decision was ultimately made by two physicians with more than 3 years of clinical experience and qualified as clinicians. It is important to note that the same injury or disease that persists without full recovery is still recognized as one injury; the same injury or disease that recurs after full recovery is recognized as two injuries. The injury assessments in this study were conducted by experienced physicians serving the national team, who evaluated the prevalence and causes of injuries without using any scales or questionnaires.

### 2.5 Statistical analysis

The experimental data were subjected to data computation through Octave 4.0.0, and secondary processed data were analyzed through SPSS 26.0 and plotted through GraphPad Prism 9.5.1. The data of the study were measured and expressed as mean ± standard deviation (mean ± SD). The injury-to-non-injury sample ratio in this study was 38:174. Impairments during the analysis were in the form of least squares classification (impairments were recorded as 1 and non-impairments as 0). The problem of the difference in magnitude between different data was taken into account during the analysis, thus all data except injury data were standardized before logistic regression and neural network modeling. In the neural network modeling process, the hidden layer activation function is a hyperbolic tangent function; the output layer activation function is SoftMax; and the error function is cross-entropy. The training set was uniformly randomly selected as 70% of the data set to train the model, and the remaining 30% of the data set was used as the test set to test the model performance and generalization ability, and the data in the test set were not involved in the process of training the model. The neural network model’s architecture and termination criteria were automated, with a minimum of 1 and a maximum of 50 units in the hidden layers. The model used the scaled conjugate gradient algorithm, with an initial Lambda value of 0.0000005 and an initial Sigma value of 0.00005. The area under the ROC curve (AUC) was used to evaluate the model’s performance (16). The confidence intervals for the models involved in the study are all 95%.

## 3 Results

### 3.1 Logistic regression

When comparing the results of 7 means logistic regression under different lag periods, the logistic regression results under no-lag period are better in terms of correct rate and significance, but this is due to the increase of non-injury samples caused by the algorithm under the lag period, and the source of the correct rate is the non-injury classification of the non-injury samples, implying that all non-injury sample predictions are correct. For the damaged samples, the correct classification rate is extremely low, with only 5 cases (out of 38) correctly classified under no lag, 1 in coupled ACWR and 2 each in EWMA and REDI; 1 case correctly classified under 5-day lag, in monotony; and 1 case correctly classified under 10-day lag, in uncoupled ACWR. The reason for this is considered more as a result of chance than model performance. Therefore, logistic regression does not perform well in the actual classification of injury and non-injury samples. This is due to the fact that there is a bias between injury and non-injury samples in the actual process, and the actual training process will reduce the appearance of injury samples through a variety of external interventions, and the logistic regression is not sensitive to the data characteristics of capturing a limited number of samples (Tables 2–4).

**Table 2:** Logistic regression results without delay.

	B	SD	Wald	*p*	Exp(B)	EXP(B) 95%CI		Accuracy
						Lower	Upper	
ACWR^a^	4.565	1.091	17.517	0.000	96.093	11.329	815.042	82.5%
UnACWR^b^	−2.432	0.859	8.024	0.005	0.088	0.016	0.473	82.1%
Diff^c^	0.811	0.459	3.130	0.077	2.251	0.916	5.528	82.1%
Monotony^d^	−0.035	0.028	1.561	0.212	0.966	0.914	1.020	82.1%
SD^e^	0.809	0.210	14.851	0.000	2.246	1.488	3.389	82.1%
EWMA^f^	5.653	1.252	20.403	0.000	285.198	24.538	3314.824	84.4%
REDI^g^	4.664	1.182	15.580	0.000	106.102	10.468	1075.432	84.4%

a Coupled acute:chronic workload ratio.

b Uncoupled acute:chronic workload ratio.

c Weekly ratio of workload change (%).

d Monotony.

e Standard deviation of weekly workload change.

f Exponentially weighted moving average.

g Robust exponential decreasing index.

**Table 3:** Logistic regression results with a 5-day delay.

	B	SD	Wald	*p*	Exp(B)	EXP(B) 95%CI		Accuracy
						Lower	Upper	
ACWR^a^	1.789	0.902	3.934	0.047	5.983	1.021	35.045	81.9%
UnACWR^b^	−1.049	0.665	2.488	0.115	0.350	0.095	1.290	81.9%
Diff^c^	1.522	0.489	9.681	0.002	4.581	1.756	11.949	81.9%
Monotony^d^	0.025	0.013	3.852	0.050	1.025	1.000	1.052	82.8%
SD^e^	0.364	0.180	4.064	0.044	1.438	1.010	2.048	81.9%
EWMA^f^	0.998	0.918	1.183	0.277	2.714	0.449	16.396	81.9%
REDI^g^	1.565	0.914	2.932	0.087	4.782	0.798	28.668	81.9%

a Coupled acute:chronic workload ratio.

b Uncoupled acute:chronic workload ratio.

c Weekly ratio of workload change (%).

d Monotony.

e Standard deviation of weekly workload change.

f Exponentially weighted moving average.

g Robust exponential decreasing index.

**Table 4:** Logistic regression results with a 10-day delay.

	B	SD	Wald	*p*	Exp(B)	EXP(B) 95%CI		Accuracy
						Lower	Upper	
ACWR^a^	−1.553	0.841	3.407	0.065	0.212	0.041	1.101	80.7%
UnACWR^b^	0.826	0.408	4.104	0.043	2.283	1.027	5.075	81.7%
Diff^c^	−0.230	0.497	0.214	0.644	0.795	0.300	2.103	80.7%
Monotony^d^	−0.300	0.025	1.394	0.238	0.971	0.924	1.020	80.7%
SD^e^	−0.283	0.169	2.790	0.095	0.753	0.541	1.050	80.7%
EWMA^f^	−1.302	0.864	2.269	0.132	0.272	0.050	1.480	80.7%
REDI^g^	−1.112	0.816	1.860	0.173	0.329	0.066	1.626	80.7%

a Coupled acute:chronic workload ratio.

b Uncoupled acute:chronic workload ratio.

c Weekly ratio of workload change (%).

d Monotony.

e Standard deviation of weekly workload change.

f Exponentially weighted moving average.

g Robust exponential decreasing index.

### 3.2 Multilayer perceptron

Comparing the results of the seven means of artificial neural networks under different lags, the seven sRPE-derived metrics multilayer perceptron models showed good classification performance in all three time series nodes (average classification accuracy: no delay >5-day delay >10-day delay, 86.5% > 79.1% > 76.6%); average AUC: no delay >5-day delay >10-day delay (0.773 > 0.645 > 0.627). In the no-lag period, the highest correct classification rate was for the coupled ACWR, but its AUC was not high, and model screening revealed that the reason originating from the excessively high number of non-injury samples in the randomized test set caused it to appear high despite its low AUC, whereas the EWMA maintained a high rate of correct classification (90%) despite its high AUC (AUC = 0.899). In the 5-day lag period, the weekly load change rate performed best, maintaining a high rate of correct classification (88.9%) despite a high AUC (AUC = 0.841). In the 10-day lag period, the overall classification model performed poorly, with monotony, a standard deviation of weekly load change, and EWMA having an AUC close to 0.5, and its classification performance was not satisfactory (Tables 5–7).

**Table 5:** Multilayer perceptron results without delay.

	Cross-entropy error		AUC		Accuracy
	Training set	Test set	Injury	Non-injury	
ACWR^a^	72.495	14.620	0.714	0.714	91.3%
UnACWR^b^	60.989	13.893	0.815	0.815	87.2%
Diff^c^	49.812	16.491	0.862	0.862	79.1%
Monotony^d^	62.575	27.331	0.715	0.715	82.5%
SD^e^	69.578	19.527	0.687	0.687	88.1%
EWMA^f^	42.075	13.104	0.899	0.899	90.0%
REDI^g^	73.951	18.405	0.721	0.721	87.2%

a Coupled acute:chronic workload ratio.

b Uncoupled acute:chronic workload ratio.

c Weekly ratio of workload change (%).

d Monotony.

e Standard deviation of weekly workload change.

f Exponentially weighted moving average.

g Robust exponential decreasing index.

**Table 6:** Multilayer perceptron results with a 5-day delay.

	Cross-entropy error		AUC		Accuracy
	Training set	Test set	Injury	Non-injury	
ACWR^a^	63.194	20.846	0.700	0.700	78.0%
UnACWR^b^	67.987	21.147	0.613	0.613	76.9%
Diff^c^	55.881	11.559	0.841	0.841	88.9%
Monotony^d^	58.756	33.116	0.619	0.619	78.1%
SD^e^	63.234	31.830	0.592	0.592	75.9%
EWMA^f^	63.908	24.061	0.630	0.630	78.7%
REDI^g^	67.470	25.753	0.520	0.520	77.1%

a Coupled acute:chronic workload ratio.

b Uncoupled acute:chronic workload ratio.

c Weekly ratio of workload change (%).

d Monotony.

e Standard deviation of weekly workload change.

f Exponentially weighted moving average.

g Robust exponential decreasing index.

**Table 7:** Multilayer perceptron results with a 10-day delay.

	Cross-entropy error		AUC		Accuracy
	Training set	Test set	Injury	Non-injury	
ACWR^a^	55.991	25.719	0.789	0.789	75.0%
UnACWR^b^	51.034	24.041	0.785	0.785	80.8%
Diff^c^	59.327	21.722	0.581	0.581	73.7%
Monotony^d^	69.811	26.063	0.553	0.553	79.2%
SD^e^	64.637	32.456	0.516	0.516	76.3%
EWMA^f^	59.171	28.781	0.503	0.503	77.8%
REDI^g^	58.129	28.768	0.659	0.659	73.5%

a Coupled acute:chronic workload ratio.

b Uncoupled acute:chronic workload ratio.

c Weekly ratio of workload change (%).

d Monotony.

e Standard deviation of weekly workload change.

f Exponentially weighted moving average.

g Robust exponential decreasing index.

Combining the performance of AUC and accuracy, the optimal two means are selected for comparison (no-lag EWMA and the 5-day lag weekly ratio of workload change), both of which have good clustering quality and perform well in capturing non-injury samples, as shown in the classification box plot. The 5-day lag weekly rate of change is better than the no-lag EWMA in capturing injury samples; however, the mean value case of the no-lag EWMA performance is slightly better than the 5-day lag weekly rate of change, but affected by the extreme values, resulting in the overall injury sample capture worse than the 5-day lag weekly ratio of workload change. For samples with abnormal load changes, it is possible that the 5-day lag weekly rate of change is more sensitive than the no-lag EWMA, probably due to the fact that EMWA has taken into account the problem of weighting the load of the time series, and still weighted the samples with abnormal load changes, reducing its sensitivity (13). However, such a practical situation often involves low injury risk, and coaches can completely combine the actual situation to make a judgment, thus for the abnormal performance of the no-lag EWMA at extreme values, this study is considered to be negligible.

Comparing the two ROC curves, the no-lag EWMA outperforms the 5-day lag weekly ratio of workload change in both the non-injury sample and the injury capture, but both perform better. Comparing the gain and benefit plots of the two they are similar in the non-injury samples, but in the injury samples, the no-lag EWMA shows better gain and benefit performance, the no-lag EWMA has already reached 100% gain compared to the no-modeled case in 50% of the data, while the 5-day lag weekly ratio of workload change has only reached 100% gain compared to the no-modeled case in 80% of the data. The no-lag EWMA achieves a 4.0-fold benefit over the no-model case at 10% of the data, while the 5-day lag weekly ratio of workload change is lower than the 4.0-fold benefit over the no-model case at 10% of the data, and at 50% of the data, the no-lag EWMA maintains a 2.0-fold benefit, while the 5-day lag weekly rate of change is already lower than the 2.0-fold benefit.

## 4 Discussion

Based on previous research, the algorithm that is theoretically most sensitive to injury risk is REDI (13). This is because the load accumulation method with natural logarithmic weighting, which REDI uses, is superior to the load accumulation method used by EWMA. Moreover, REDI also considers the issue of missing data. By correlating the next week’s injury with the load of the current week at different time lags, the correlation between injury and load can be increased (12). This is because the occurrence of injury is delayed and there is an incubation period. Therefore, this study hypothesizes that correlating the next week’s injury with the load of the current week (with a 5-day lag) using REDI’s ACWR calculation method would yield the highest correlation between injury and load. However, the research results of this study are different from the expected hypothesis.

Among the different models (logistic regression vs. neural network) at different time lags (no lag vs. 5-day lag vs. 10-day lag), EWMA without any time lag was found to be the most sensitive indicator for detecting injury risk. According to the results of logistic regression, only EWMA and REDI without any time lag showed ideal classification performance (Accuracy_EWMA&REDI_ = 84.4%, *p* < 0.001). The results of the neural network showed that EWMA and REDI without any time lag exhibited ideal classification performance (Accuracy_EWMA_ = 90.0%, Accuracy_REDI_ = 87.2%, ROC_EWMA_ = 0.899, ROC_REDI_ = 0.721). These results are consistent with previous studies. Under the same model, the results without any time lag were superior to those with other time lags (5-day lag and 10-day lag), which differs from previous research. We speculate that this discrepancy may be due to project differences. There were also differences between the results of different models (logistic regression vs. neural network). A common finding is that the accuracy of model classification decreases with longer time lags. However, the ACWR calculation method differs at different time lag nodes. Additionally, the results of logistic regression were more stable, while the neural network showed the best performance without any time lag, but its performance was worse than logistic regression at other time lag nodes. The reason for this may be related to the proportion of injuries, which will be explained in detail later in the text.

**Figure 1:**
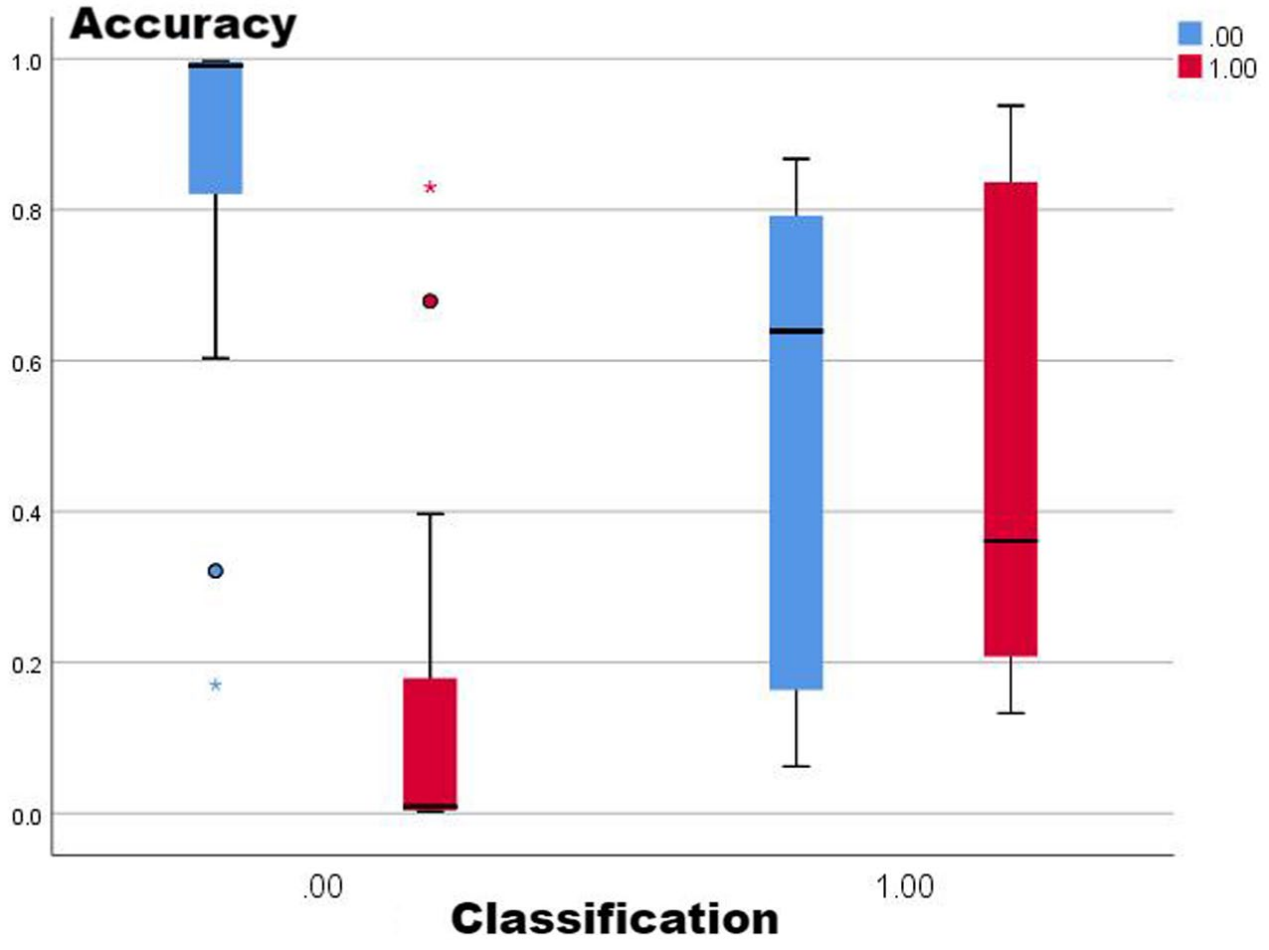
Sorted box plot from EMWA without delay.

**Figure 2:**
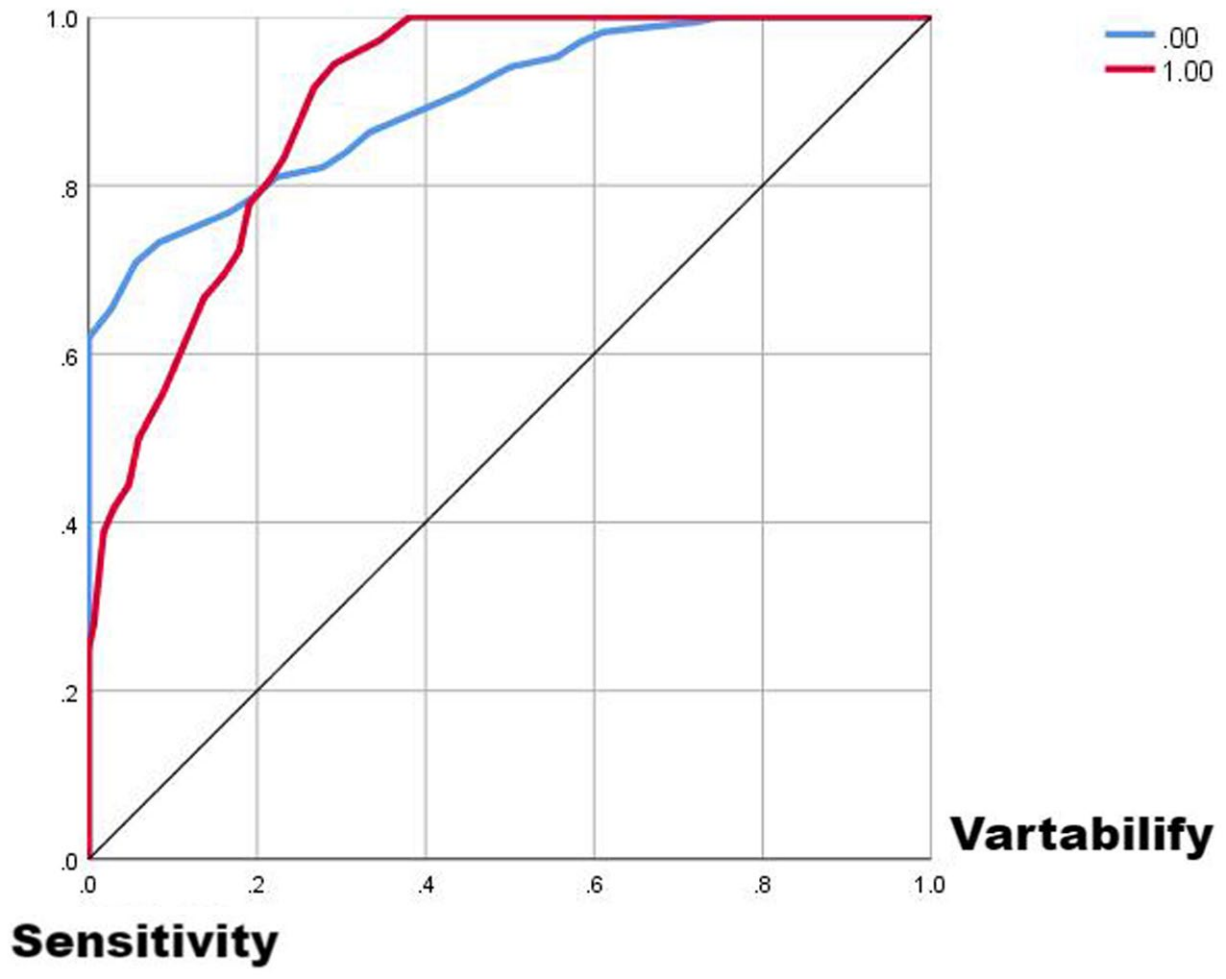
ROC curve plot from EMWA without delay.

**Figure 3:**
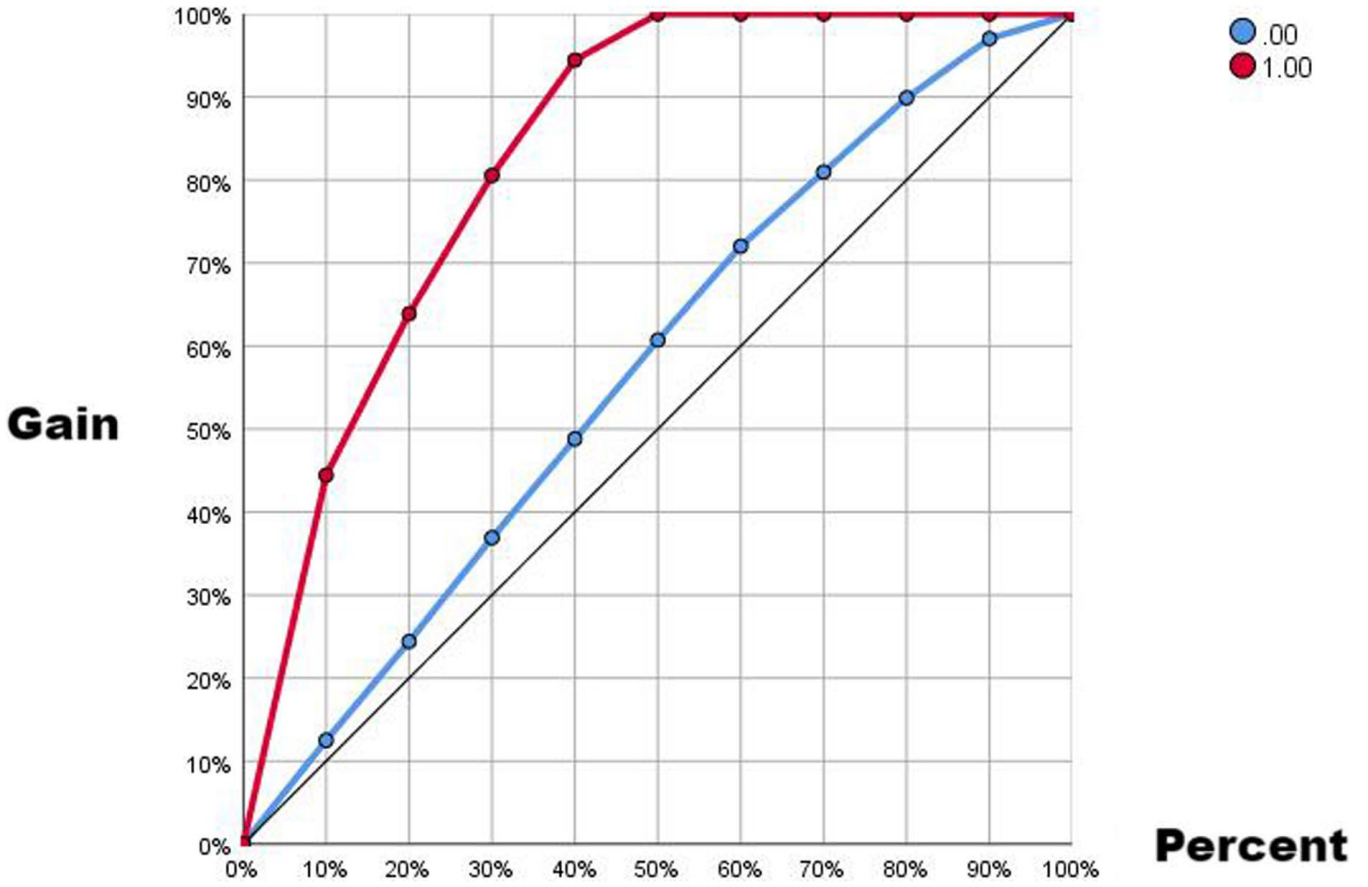
Gain plot from EMWA without delay.

**Figure 4:**
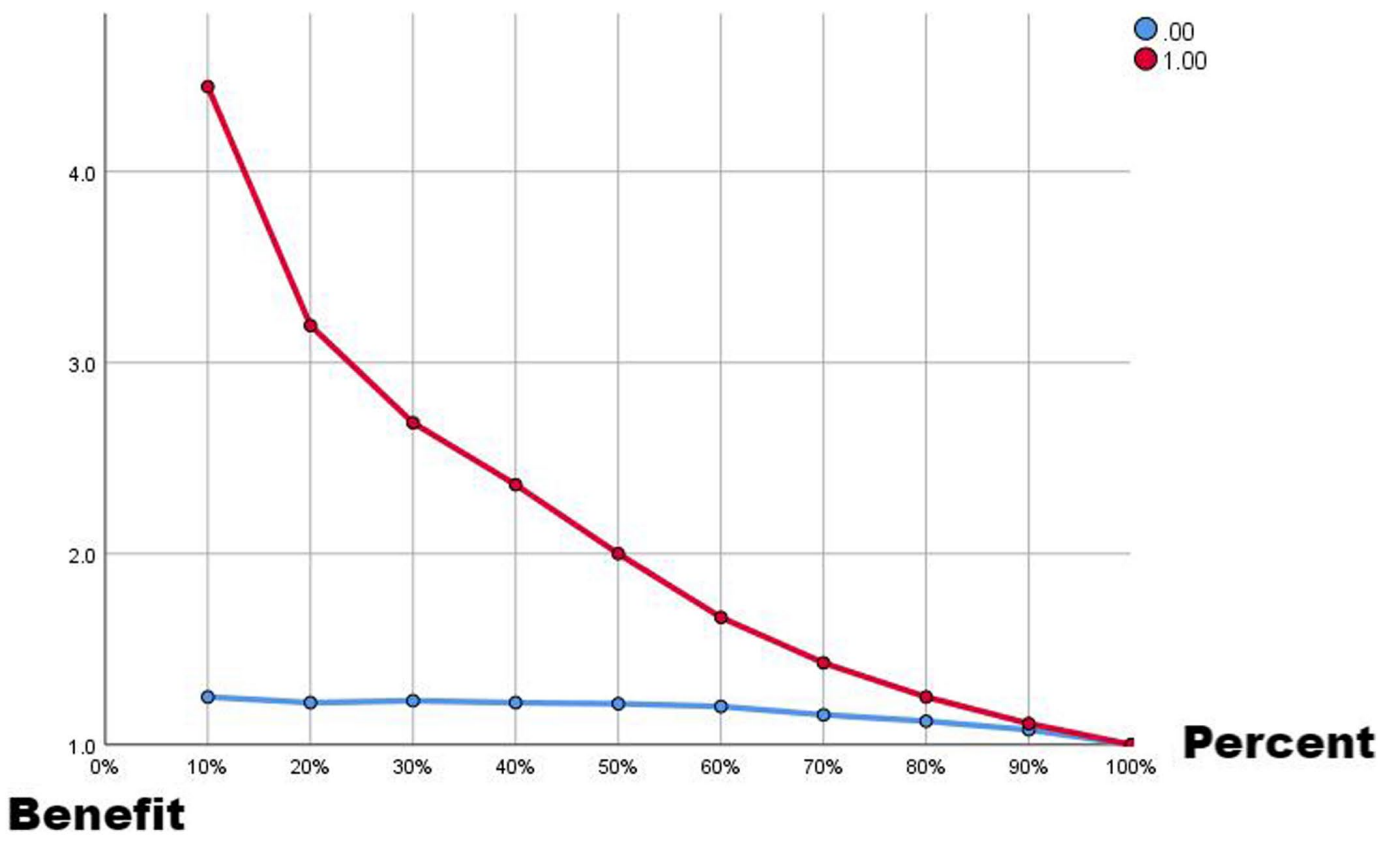
Benefit plot from EMWA without delay.

**Figure 5:**
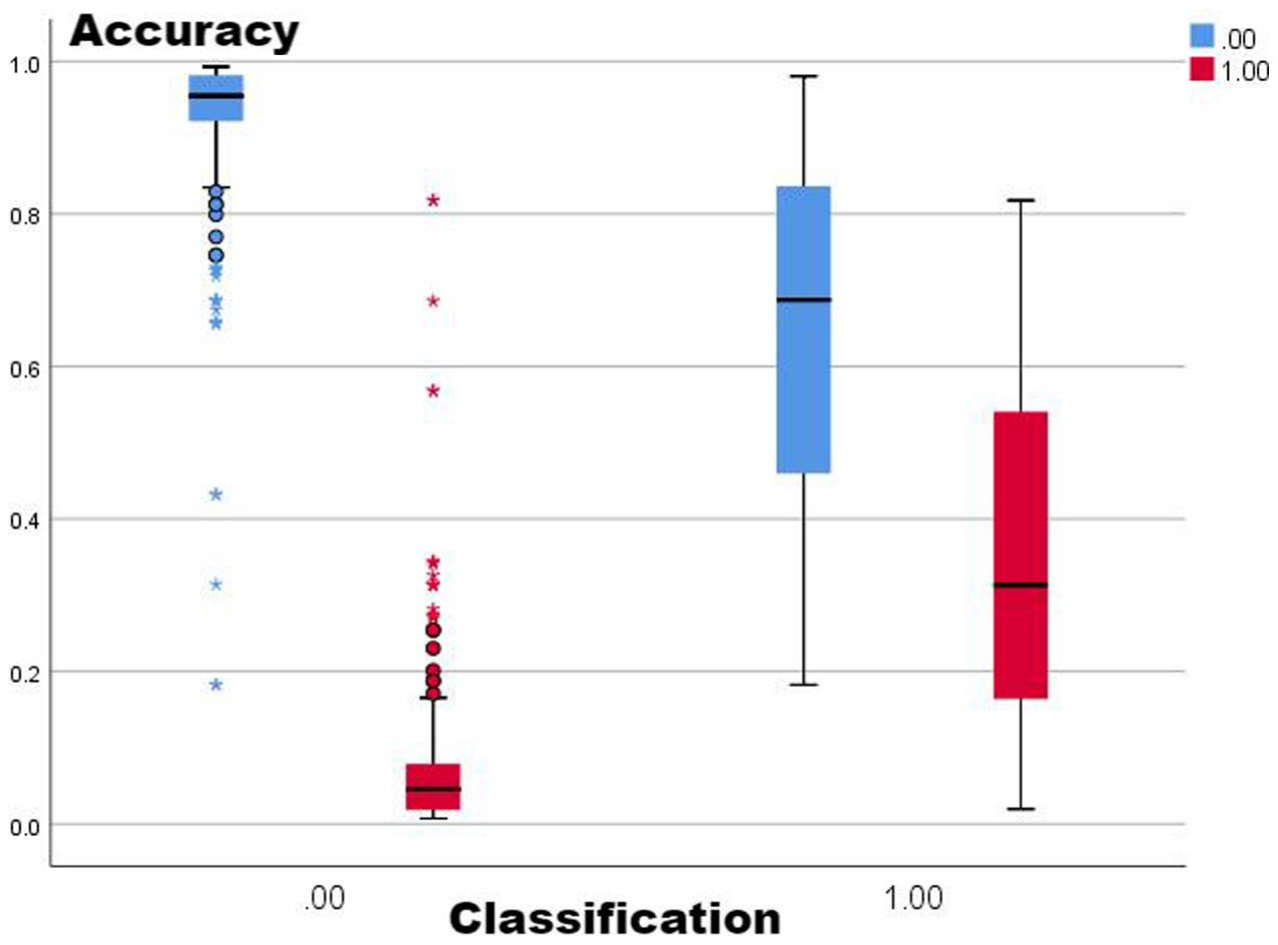
Sorted box plot from Diff with a 5-day delay.

**Figure 6:**
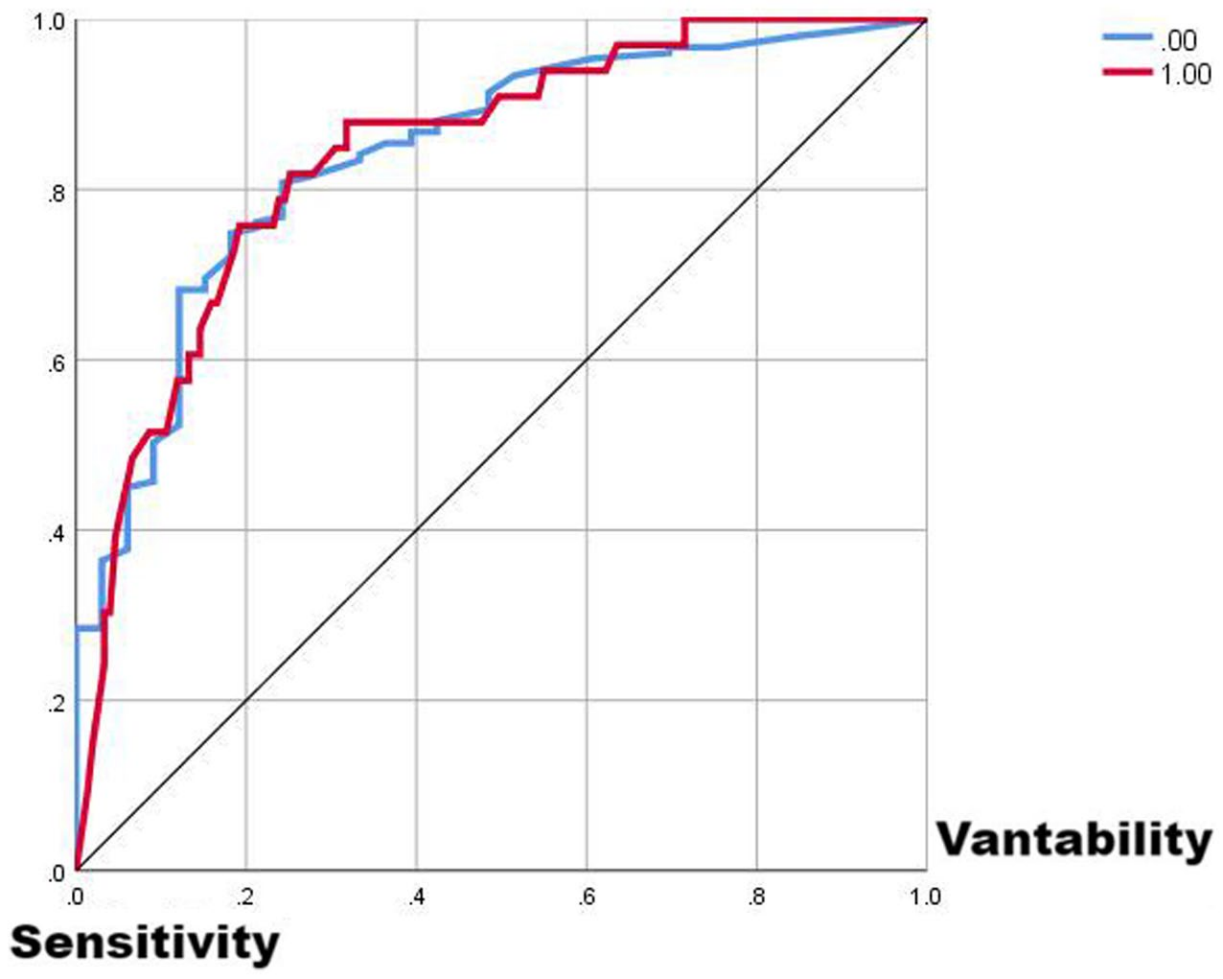
ROC curve plot from Diff with a 5-day delay.

From Figures 1–5, it can be observed that the two models with the best injury classification performance have extremely high accuracy in classifying non-injury cases, but the classification accuracy for injury cases is between 30 and 40%. The accuracy of EWMA without any time lag is slightly better than that with a 5-day lag. This result indicates that curling athletes do indeed have a problem of load accumulation in their training, which needs to be considered in load monitoring (Figure 6).

### 4.1 Data bias

**Figure 7:**
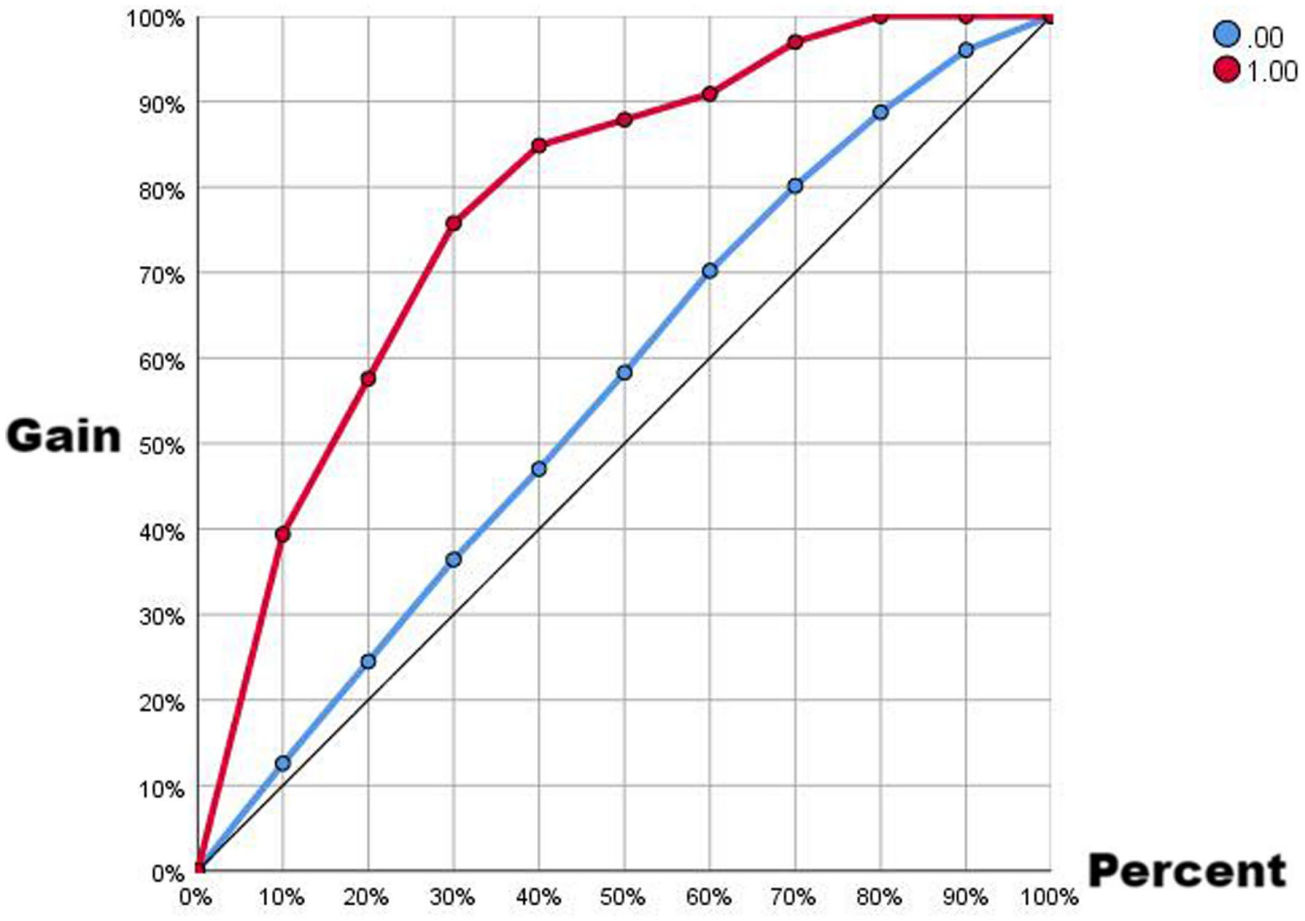
Gain plot from Diff with a 5-day delay.

Logistic regression and generalized estimating equations have been used in most of the studies on ACWR, and quadratic regression and generalized linear models have been used in a few studies. In the analysis of damage classification, the significant challenges are damage data bias, a large amount of non-damage data, and a small amount of damage data. This puts higher demands on data analysis and requires that the analysis method captures the features of damaged data more accurately. In conjunction with the findings of this study, the results of the neural network were overall better than those of logistic regression, supporting the results that the adoption of deep learning for deep data mining and capturing data features in the field of sports science has been a driving force for progress in practice (17) (Figure 7).

### 4.2 Workload accumulation

**Figure 8:**
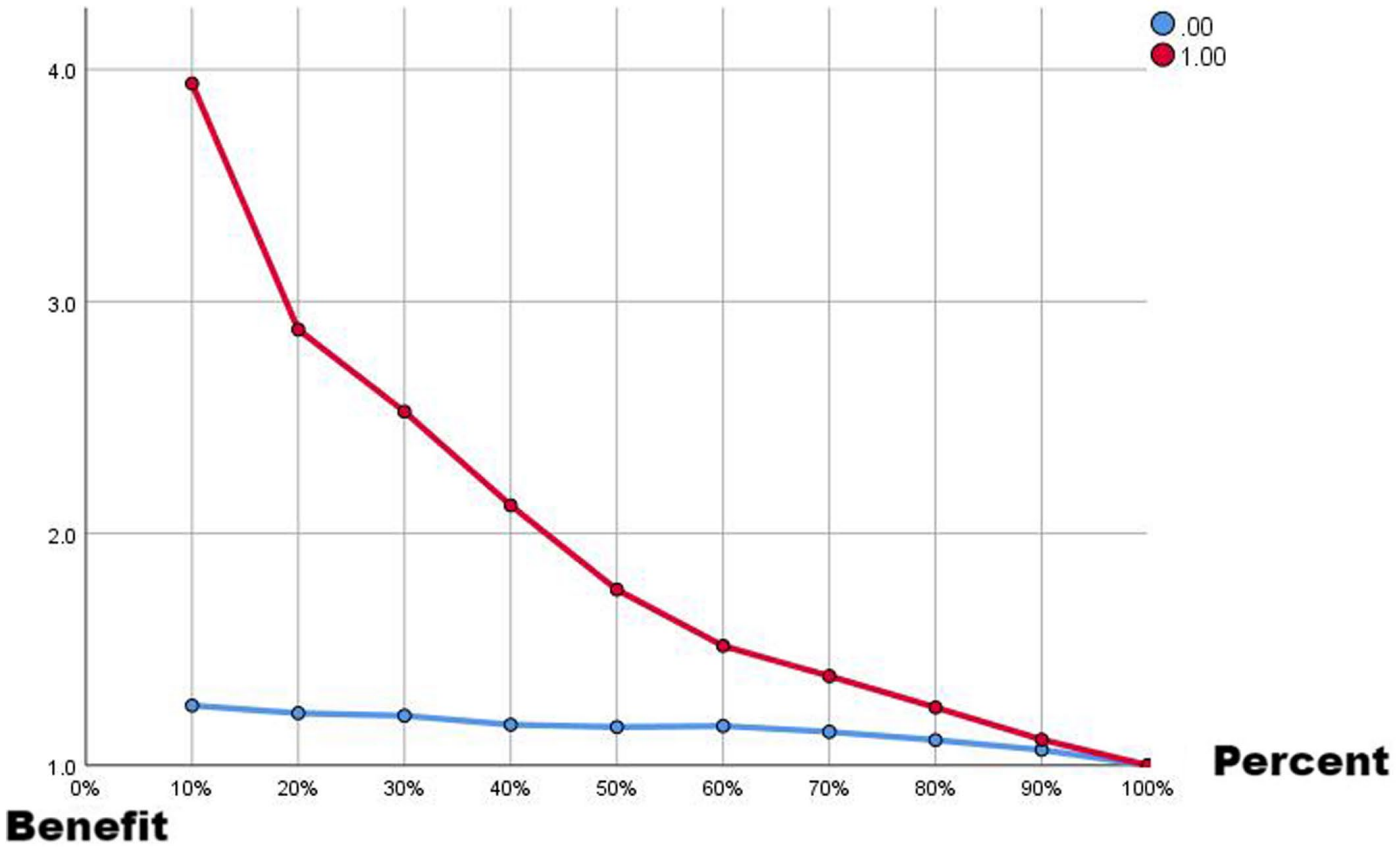
Benefit plot from Diff with a 5-day delay.

The problem of load time series superposition, the effect of past loads on current loads, has been neglected in past load monitoring studies. Although it is not necessary to take this into account in some load monitoring tools, especially immediate state evaluation, it is necessary to take this into account in the use of sRPE. The results of this study support that EMWA’s load-weighting approach is the optimal result (11). Moussa proposed the REDI approach to solving the problem of missing load time series (rest day loads are recorded as 0 A.U.) (13) and argued that REDI would outperform the EWMA in the presence of missing data but the results of the present study do not support this view. Despite the presence of missing data, the performance of EWMA with different lags outperforms the performance of REDI (Figure 8).

### 4.3 Hysteresis period

Previous researchers have argued that there is a lag in the emergence of load (12), and it is true that for the weekly ratio of workload change, the consideration of the lag period is valuable, and among no lag, 5-day lag, and 10-day lag, a weekly ratio of workload change with a 5-day lag performs the best, but the results of the analysis of other means with no lag were better than the results of the 5-day and 10-day lag. Therefore, for the other means, the risk of injury is studied in the relationship with loading before, there is no need to consider the lag period.

## 5 Conclusion

In capturing load risk by sRPE, EWMA without delay is a more sensitive indicator for detecting damage risk, which has practical significance by taking into account the load time series weights and the lag period of damage occurrence. It is suggested that the time decay of load time series weights and the lag time of damage occurrence should be considered according to the actual situation when sRPE is used for load monitoring in the future.

## Data availability statement

The data analyzed in this study is subject to the following licenses/restrictions: the datasets used or analyzed during the current study are available from the corresponding authors on reasonable request. Requests to access these datasets should be directed to wujunqi567@foxmail.com.

## Ethics statement

The studies involving humans were approved by Sport Science Experiment Ethics Committee of Beijing Sport University, No. 2023283H. The studies were conducted in accordance with the local legislation and institutional requirements. The participants provided their written informed consent to participate in this study.

## Author contributions

JW: Conceptualization, Formal analysis, Investigation, Methodology, Software, Writing – original draft. FZ: Data curation, Supervision, Writing – original draft. CL: Conceptualization, Resources, Supervision, Writing – review & editing.
